# Evaluation of spray-dried eggs as a micronutrient-rich nutritional supplement

**DOI:** 10.3389/fnut.2022.984715

**Published:** 2022-09-02

**Authors:** Philip Pirkwieser, Silke Grosshagauer, Andreas Dunkel, Marc Pignitter, Bernard Schneppe, Klaus Kraemer, Veronika Somoza

**Affiliations:** ^1^Leibniz Institute for Food Systems Biology at the Technical University of Munich, Freising, Germany; ^2^Department of Physiological Chemistry, Faculty of Chemistry, University of Vienna, Vienna, Austria; ^3^OVOBEST Eiprodukte GmbH & Co. KG, Neuenkirchen-Vörden, Germany; ^4^Sight and Life Foundation, Basel, Switzerland; ^5^Department of International Health, Johns Hopkins Bloomberg School of Public Health, Baltimore, MD, United States; ^6^School of Life Sciences Weihenstephan, Technical University of Munich, Freising, Germany

**Keywords:** food quality and safety, nutritional supplement, nutrient adequacy, chicken egg, spray-drying

## Abstract

Regular consumption of hen eggs can help to prevent deficiencies of essential nutrients, such as essential amino acids, vitamin A and E or trace elements zinc and selenium, for vulnerable populations. This study focused on assessing the nutritional value of spray-dried eggs, favored by their manufacturability, storability and ease of addition to (complementary) foods. Using a wide range of analytical techniques, we recorded and compared the nutrient profiles of commercially produced pasteurized whole eggs and their respective powder samples spray-dried at 160°C. Important nutrients that were not significantly affected by spray-drying include total fat content, several amino acids, α- and δ-tocopherol, lutein, zeaxanthin, essential trace elements and cobalamin. The most notable mean losses were found for unsaturated fatty acids, e.g., linoleic (by −38.7%, from 4.11 ± 0.45 to 2.52 ± 0.75 g/100 g DM) and linolenic acid (by −60.8%, from 0.76 ± 0.05 to 0.30 ± 0.04 g/100 g DM). Despite recording significant retinol losses in two out of three batches, the overall low reduction of −14% recommend spray-dried eggs as a valuable source of vitamin A. A daily intake of spray-dried egg powder corresponding to one medium sized egg meets dietary reference values for children, e.g., by 100% for vitamin E, by 24% for retinol, by 61% for selenium and by 22% for zinc. In conclusion, even though a dry weight comparison favors supplementation with pasteurized whole eggs, our results demonstrate a high potential for spray-dried eggs as nutritional supplement. However, the spray-drying process should be optimized toward higher retentions of unsaturated fatty acids and retinol.

## Graphical Abstract

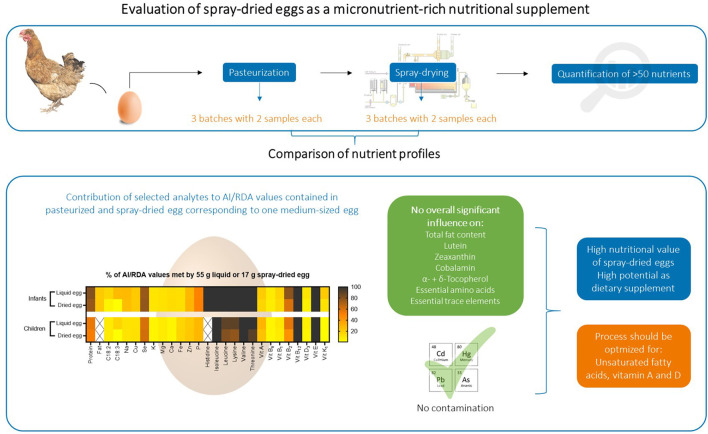

## Introduction

Hen eggs are appreciated globally as inexpensive, nutritious and technologically versatile foods. Steadily increasing production numbers over the past years, approaching 80 million tons per year, and a thereby calculated global average per capita of ~161 eggs demonstrate their growing demand. Naturally, consumption greatly varies regionally, with annual per capita numbers ranging from 300 g in several African countries up to 19.1 kg in Japan (1, 2). The fact that whole egg is a valuable source of protein is made clear above all by its defined biological value of 100% (3). With this biological value, egg serves as a reference value for other foods and indicates how efficiently the dietary protein can be utilized as the body's own protein. With the exception of vitamin C, hen eggs contain all vitamins and represent an important source of nutrients. All fat-soluble vitamins A, D, E and K are found in the egg's yolks, with an average content per 100 g egg of 276 μg vitamin A (as retinol), 2.3 mg vitamin E (as total tocopherols), 2.9 μg vitamin D and 8.9 μg vitamin K. With these contents, eggs make an important contribution to meeting daily vitamin requirements of 0.8–1 mg of vitamin A, 12–15 mg of vitamin E, 20 μg of vitamin D and 60–70 μg of vitamin K (4).

The increasing knowledge on the efficient enrichment of fat-soluble vitamins in egg yolk ensued the idea of using hen eggs as an effective measure against dietary deficiencies, especially in developing countries (5, 6). For instance, this approach could be of importance for sub-Saharan African regions, where vitamin A deficiency is widespread and leads to a high prevalence of vision losses (7). Promising results on the supplementation with eggs could already be shown in intervention studies, e.g., regarding child growth by Iannotti et al. (8), where the addition of one egg a day to complementary foods of older infants reduced stunting and underweight by 47 and 74%, respectively. However, nutrition surveys from Europe and America also revealed that a sufficient vitamin E intake should not be taken for granted in developed countries (9). Since eggs are consumed regularly by a majority of the worldwide population, they can make an important contribution to meeting the requirements of fat-soluble nutrients. Yet, implementing strategies of increasing the consumption of hen eggs as sources of valuable essential nutrients requires both knowledge of the efficiency and technological implementation of nutrient fortification and consideration of economic and cultural factors. Particularly in areas where malnutrition is part of everyday life, availability and affordability of eggs is severely limited, with only two eggs monthly per person (10).

For technological as well as safety reasons, dried egg in all its variants—whole egg powder, egg yolk powder, egg white powder—is increasingly used in the food industry. In contrast to dried eggs, shell eggs are not only fragile, they also require far more storage space, are more susceptible to microbial spoilage and carry a higher risk of salmonella (11). For food technology use, shell eggs are therefore often homogenized and pasteurized, which reduces the risk of salmonella transmission. Subsequent drying of the eggs also provides the advantage of reducing the water content to a minimum, thereby achieving a longer shelf life. Another benefit is that dried eggs offer a more convenient utilization, especially on a large industrial scale. Above all, drying reduces both weight and volume, resulting in a concentration of nutrients (12), suggesting a significant potential as nutritional supplement to effectively prevent malnutrition in vulnerable populations.

Another crucial point to consider for nutritional supplements is consumer acceptance—fortified foods or alternative sources of nutrients are often restrained by off-flavors (13, 14), whereas eggs are widely accepted and versatile.

Spray-drying is a common technique for dehydration of foods, in which droplets of the liquid food are formed by, e.g., a nozzle or an atomizer, and then transferred to a drying chamber, where incoming hot air causes the water to evaporate. The high-throughput process guarantees relatively low heat-spoilage and yields products of constant, pre-determined characteristics, ready for packaging (15). Although further processing of eggs to powder *via* spray-drying (usually at 160° or 180°C) is gaining more and more importance in the food industry, mirrored in several studies on the functional properties after drying (16, 17), little knowledge of how this process actually affects the eggs' entire nutrient profile exists. This obviously represents a major knowledge gap regarding the use of spray-dried eggs as nutritional supplement. A recent study by Abreha et al. (18) compared the fat and protein content of egg powder produced from eggs of two chicken breeds and attributed a high potential to its use as supplementary food. However, even though several key parameters were determined, analyses of the impact of spray-drying on, e.g., the contents of fatty acid and vitamins or the formation of peroxides were not included. Other reported influences of spray-drying on nutrients include losses of carotenoids, e.g., a reduction of lutein during pasteurization and further deterioration after spray-drying in egg yolk from carotenoid-enriched eggs (19). By contrast, Caboni et al. (20) found no significant difference in retinol contents of pasteurized whole egg and spray-dried egg powder. Additional storage experiments of these whole egg samples yielded only a slight decrease of fat-soluble vitamins at 4°C for 12 months, as opposed to a significantly greater deterioration when stored at 20°C over the same period of time (20).

Nevertheless, fortified hen's diet, a limited number of analytes or a spray-drying process incomparable to industrial scale reported impede general statements on the suitability of the spray-drying process for eggs from a nutritional point of view. To further elucidate the viability of spray-drying as a suitable method for the use of eggs as nutritional supplements, we performed a comprehensive analysis of food quality and safety related key analytes, using commercially produced pasteurized whole eggs and their respective spray-dried egg powder. Besides investigating the desirable preservation of nutrients, including vitamins A, D, E and unsaturated fatty acids, a putative enrichment of anti-nutrients or potential toxicants, such as non-essential trace elements, was monitored as well. Since higher temperatures and contact with oxygen favor lipid oxidation, the peroxide value was also part of the chemical-analytical examination. In this regard, e.g., Verardo et al. (21) found a twofold increase in peroxide content from 2.6 ± 0.3 meq O_2_/kg fat in pasteurized eggs to 5.3 ± 0.4 meq O_2_/kg fat in spray-dried samples. Ultimately, the purpose of the here presented work was to display whether spray-dried eggs have the potential to contribute to daily nutrient requirements as a supplement to fat-based complementary foods or whether the consumption of pasteurized whole eggs would be more beneficial to meet the dietary reference intakes (DRI). Therefore, the effect of spray-drying was studied in-depth for key nutrients of relevance regarding the use of spray-dried eggs as nutritional supplement. Obtained results were translated to their contribution to reference intakes [Adequate Intakes (AI) and Recommended Dietary Allowances (RDA)] for infants and children. The data presented herein forms the most complete assessment on the effect of industrial spray-drying on the nutritional value of eggs to date. The findings should help improve the spray-drying process for the subsequent use of eggs as high quality nutritional supplement in the future, especially as an effective measure against malnutrition in vulnerable populations of low- and middle-income countries.”

## Materials and methods

### Chemicals

Chemicals and reagents used for sample preparation, instrument calibration and analysis were the following. Ultrapure water of resitivity >18.2 MΩ^*^, ethanol 96% (VWR), ethanol (absolut Emsure^®^, Merck), methanol (HPLC grade ≥ 99.9%, Sigma-Aldrich), n-hexane (Emplura^®^ ≥ 95%, Merck), toluene (for analysis, Emsure^®^ ACS Merck), chloroform (≥99% for synthesis, Carl Roth), tert.-Butylmethylether (EMSURE^®^ ACS, for analysis, Merck), acetic acid (glacial, EMSURE^®^ ACS Merck), nitric acid (ROTIPURAN^®^Supra 69%, Carl Roth), hydrochloric acid (≥37%, ACS, Sigma-Aldrich), hydrogen peroxide (trace metal grade, Sigma-Aldrich), pyrogallol (ACS reagent, ≥99%, Sigma-Aldrich), sodium sulfate (anhydrous powder, ≥99% ACS reagent, Sigma-Aldrich), sodium sulfide (reagent grade, Sigma-Aldrich), sodium-L-ascorbate (crystalline, ≥98%, Sigma Aldrich), sodium hydroxide (≥85% p.a., Carl Roth), monopotassium phosphate (ACS, ≥9% Carl Roth), barium chloride-dihydrate (Emsure^®^, ACS Merck), iron(II)sulfate-hydrate (86.0–89.0%, FeSO_4_ basis) and iron(III)chloride-hexahydrate (97%, ACS, Sigma-Aldrich), sodium chloride (≥99.5%, p.a. ACS, Carl Roth), sodium methoxid (purum, ≥97.0%, Sigma-Aldrich) and xylenol orange tetrasodium salt (ACS, Sigma-Aldrich). Standard solutions retinol (powder), (±)-α-tocopherol, (±)-γ-tocopherol and (±)-δ-tocopherol, Butylhydroxytoluene, zeaxanthine (≥95%), methyl heptadecanoate (all HPLC standards, Sigma-Aldrich), lutein (≥96%, Merck), Instrumental Calibration Standard 2 and Alternate Metals solution (PerkinElmer), single standard solutions for mercury (PurePlus, PerkinElmer) and phosphorus (Aristar, VWR). The internal standard solution contained Sc, Rh, Ge and Re.

### Instrumentation

The following instruments haven been used in this study. Ultrapure water system Arium 611 UF (Sartorius), centrifuge 5804 R and 5810 R (Eppendorf AG), concentrator 5301 (Eppendorf AG), gas chromatograph GC-2010 Plus, AOC 20is (Shimadzu), incubator INCU-Line ILS4 (VWR International), photometer Infinite M200 (Tecan), rotavapor R210 (Büchi), reflux condenser DIN NS29/32; 400 mm (Lenz Laborglasinstrumente), (U)HPLC-DAD UltiMate 3000RS (Dionex), Exion LC UHPLC system (Sciex, Darmstadt, Germany) connected to a QTRAP 6500^+^ mass spectrometer (Sciex) using atmospheric pressure chemical ionization (APCI), bead-beater homogenizer (Precellys Evolution, Bertin Technologies), ICP-MS Nexion 5000 (PerkinElmer), microwave digestion system Multiwave 5000 (Anton Paar) and ultrasonic bath USC TH (VWR International). The respective application of these instruments is given in the following Methods section.

### Methods

#### Study design and egg treatment

In order to investigate the influence of spray-drying on the nutrient profile of hen eggs, samples from three different batches of pasteurized whole egg as well as their respective spray-dried whole egg powder were analyzed. Processing of the eggs was kindly conducted by OVOBEST Eiprodukte GmbH & Co KG as follows: Fresh eggs were washed, automatically broken, filtered and homogenized. The contents were then conveyed to pasteurization at 62.2°C. Since the eggs were subsequently to be processed into powder, they were additionally pre-dried in the evaporator, yielding an average dry matter content of 30.3%, as opposed to 23.5–24% usual for shell eggs. At this stage, two samples were taken from each batch. For the powder, the liquid whole eggs were taken to the spray-drying plant (Sanovo Technology Group), where they were finely atomized through a nozzle and the resulting spray cone of fine egg droplets was sprayed into the box of the dryer. The inflow of hot air at 160°C caused the water to evaporate rapidly and the dry particles to fall to the bottom. The temperature of the outflowing air was kept between 80 and 90°C. The applied pressure ranged between 10,000 and 14,500 kPa, adjusted depending on outflowing air temperature and sample water content. The obtained powder was transported out of the spray-drying system, where two samples were taken from each batch and then aliquoted and stored at−80°C until analysis. An additional batch was prepared with an inflow temperature of 180°C and pressure ranging between 12,500 and 16,000 kPa, in order to monitor the influence of increased temperature on selected analytes. A simplified flow sketch of this procedure is illustrated in Figure S1 in Supplementary material.

#### Analytical methods

For fat extraction, the well-known and still commonly used (22) method according to Folch et al. (23) was chosen. The fat content was determined by differential weighing and the extracted fat was used immediately afterwards to measure the peroxide value.

Fatty acids were determined in the form of their fatty acid methyl esters as described by Aparicio and Aparicio-Ruíz (24) and Lall et al. (25) The measurement was performed by means of Gas chromatography (GC) followed by flame ionization detection (FID).

For quantitation of vitamin E, tocopherols were first extracted from the liquid egg and dried egg and separated by HPLC with reversed phase chromatography (RP). Here, separation of α-tocopherol, γ-tocopherol as well as δ-tocopherol was achieved. The β-homolog, which occurs in comparatively smaller amounts than the other homologs, could not be separated and thus quantitatively determined. Detection was performed by UV at a wavelength of 295 nm. The preparation of the sample was based on the description of Meluzzi et al., (26) which was modified as follows. From the thawed liquid egg, 5 g or, from the egg powder, 1–2 g were weighed into a 100 ml Erlenmeyer flask and used for extraction and saponification with 30 ml ethanol:potassium hydroxide solution (1:1 vol/vol). The samples were gassed with nitrogen and stirred for 30 mins. After the addition of 20 ml hexane (+1 g/L butylhydroxytoluene) and 20 ml of a saturated potassium dihydrogen phosphate (KH_2_PO_4_) solution, the sample was stirred for an additional 5 mins and transferred to a separating funnel. After separation, the organic phase (~20 ml) was collected in 50 ml centrifuge tubes. The aqueous phase in the Erlenmeyer flask was transferred back to the funnel and extracted again with 20 ml hexane (+1 g/L butylhydroxytoluene). The combined organic phase (about 40 ml in total) was concentrated over nitrogen. After complete constriction, the sample was taken up in 2 ml of ethanol and transferred *via* syringe filter into an HPLC vial. The tocopherols were then measured by HPLC-UV with the following conditions: Kinetex 5 μm EVO C18, 150 × 4.6 mm column, H_2_O and methanol as mobile phases, 0.5 ml/min flow rate, 20 μl injection volume, a temperature of 10°C and detection by diode array detector (Dionex DAD-3000).

For quantification of the carotenoids lutein and zeaxanthin, LC–MS/MS analysis was performed using an Exion LC UHPLC system (Sciex, Darmstadt, Germany) connected to a QTRAP 6500^+^ mass spectrometer (Sciex) using atmospheric pressure chemical ionization (APCI) in positive mode. The UHPLC systems consisted of two Exion LC AD pumps, an Exion LC degasser, an Exion LC AC column oven, an Exion LC AD autosampler, and an Exion LC controller. Chromatographic separation was achieved using a C30 Carotenoid column (250 × 4.6 mm, particle size 3 μm; YMC Europe GmbH, Dinslaken, Germany) and methanol/methyl tert-butyl ether(MTBE)/water (81/15/4, v/v/v, solvent A) and methanol/ MTBE/water (7/90/3, v/v/v, solvent B) as solvents at 40°C column temperature. The following binary gradient was applied using a total flow rate of 0.4 ml/min: 0 min 0% B, 5 min 0% B, 20 min 20% B, 22 min 100% B, 25 min 100% B, 28 min 0% B, 35 min 0%B. The mass spectrometer was operated in MRM mode, zero grade air served as the nebulizer (55 psi) and as nebulizer gas (450°C, 65 psi) and nitrogen as a curtain (35 psi) as well as collision gas. Data acquisition and instrumental control was performed using Analyst 1.6.3 software (Sciex), while quantitative data evaluation was completed with MultiQuant software (Sciex, version 3.02). To optimize the MS/MS detection for high sensitivity quantification, standard solutions of the analytes were infused into the MS instrument by the integrated syringe pump at a flow rate of 7 μl/min. The optimization of ion path parameters yielded the following values, that were applied for lutein and zeaxanthin, respectively: quantifier transition: Q1 m/z 569.3, Q3 m/z 569.2, dwell time 25 ms, declustering potential (DP) 106 V, collision energy (CE) 9 V, collision cell exit potential (CXP) 16 V, Qualifier transition: Q1 m/z 569.3, Q3 m/z 135.0, dwell time 25 ms, DP 106 V, CE 29 V, CXP 16 V. For sample preparation, egg samples were weighed into bead beater tubes (2 ml, CKMix, Bertin Technologies, Montigny Le Bretonneux, France) followed by addition of 500 μL extraction buffer containing a 1+1 mixture of MTBE and hexane and 100 μL of absolute ethanol containing butylated hydroxytoluene (BHT, 500 mg/L). Homogenization and extraction was performed using a bead-beater homogenizer (9,000 rpm for 3 × 30 and 30 s breaks). After equilibration (60 min, 10°C) and centrifugation (15 min, 12,000 rpm), the supernatant was membrane-filtered (Minisart RC 15, 0.45 μM, Sartorius, Goettingen, Germany) and directly injected (1 μl) into the LC-MS/MS instrument.

Retinol in whole egg samples was determined using the method of Pignitter et al. (27) For this purpose, the sample was saponified with KOH, followed by the addition of sodium ascorbate and sodium sulfide, extraction with hexane and measurement by HPLC-UV at 325 nm.

Regarding information on lipid oxidation, measurement of the peroxide value was considered sufficient, as the eggs used for this study did not have a long storage time and were analyzed soon after the laying date or after further processing into whole egg powder. The peroxide content in foods with a high fat content, such as cooking oil, is often determined by the Wheeler (28) method. Since hen eggs have a fat content of ~10 g/ 100 g and thus a larger sample quantity would be required to extract enough fat, the peroxide number was determined using the FOX (ferrous oxidation-xylenol orange) assay (29).

For quantitative multi-elemental analysis by ICP-MS, approximately 250 mg of homogenized sample were prepared *via* microwave assisted digestion, using a solution consisting of 2 ml ultrapure water, 3 ml HNO_3_ (69%) and 1 ml H_2_O_2_, heated at 190°C for 40 min. After digestion, samples were transferred to 25 ml volumetric flasks and filled with ultrapure water. Calibration standards were prepared from multi-elemental standards Instrumental Calibration Standard 2 and Alternate Metals as well as from single standard solutions for mercury and phosphorus. The internal standard solution contained Sc, Rh, Ge and Re. Reference materials used for method validation were skimmed milk powders ERM^®^-BD150 and ERM^®^-BD151 [European Commission, Joint Research Centre (JRC)] as well as TYG093RM [Heavy Metals in Infant Cereal (rice based), FAPAS].

Analysis of the remaining parameters were conducted by accredited laboratory Chemisches Institut Burkon, Nuremburg (Germany) based on ISO standards. In detail, dry matter was determined using method L 06.00-3: 2014-08^*^, amino acids according to methods EU 152/2009, ISO 13903: 2005, using IC-UV. For the vitamin B group, the following methods were used: BS EN 14122: 2014 mod. by LC-FLD for B_1_, EN 14152: 2006 mod. using LC-FLD for B_2_, EN 14164: 2014 by LC-FLD for B_6_ and J.AOAC 2008, vol 91 No 4 by LC-UV/DAD, for B_12_. Vitamin D_3_ analysis was conducted according to EN 12821: 2009-08 by LC-DAD and vitamin K_1_ according to EN 14148: 2003 by LC-FLD.

#### Statistical evaluation

Three batches of the pasteurized whole egg samples and the respective spray-dried samples were analyzed, each with two samples per batch that were then prepared and measured at least in duplicates. The measured data was prepared in Microsoft Excel (Office 365). Any outliers were excluded from the subsequent analysis with GraphPad Prism (9.3.1) using the Nalimov outlier test. Using a single factor analysis of variance as a non-parametric test (Welch's ANOVA), statistical significance (*p* < 0.05) was tested and reported in terms of mean (MW) ± standard deviation (SD). Figures were generated using GraphPad Prism (9.3.1).

## Results and discussion

The obtained data was prepared and illustrated in three ways for the following discussion: (I) In mass of a nutrient per 100 g sample for comparing with results found in literature. (II) Absolute masses of a nutrient found in the respective masses of liquid egg and spray-dried egg powder corresponding to one medium-sized 60 g egg, given in mass per egg, where the relation was determined by measuring dry matter contents of all samples, thus revealing any losses that may have occurred during further processing. (III) How the consumption of spray-dried egg powder corresponding to the mass of one egg helps to achieve dietary reference intakes for infants aged 7–12 months and children aged 1–8 years, calculated in percentage. In addition to the illustrated and discussed results, all obtained mean values are summarized in Supplementary Tables S1, S2.

### Dry matter

The dry weight of shell eggs normally ranges between 23 and 24% (30), but as the eggs used for this study were concentrated after pasteurization using a vacuum evaporator, an average dry matter of 31.5% was obtained, which was also taken into account in the interpretation and comparison with results of other studies. The mean values for liquid eggs were 32.4, 29.3 and 32.9% for the three investigated batches, respectively, and 97.7, 97.3 and 97.5% for the respective spray-dried samples. These results lead to factors of 3.02, 3.33 and 2.97 for calculating from fresh weight (FW) to dry matter (DM) for the individual batches and to masses of 55 g pasteurized whole egg and 17 g spray-dried egg equaling one 60 g medium-sized whole egg.

### Total fat content and fatty acid distribution

**Figure 1:**
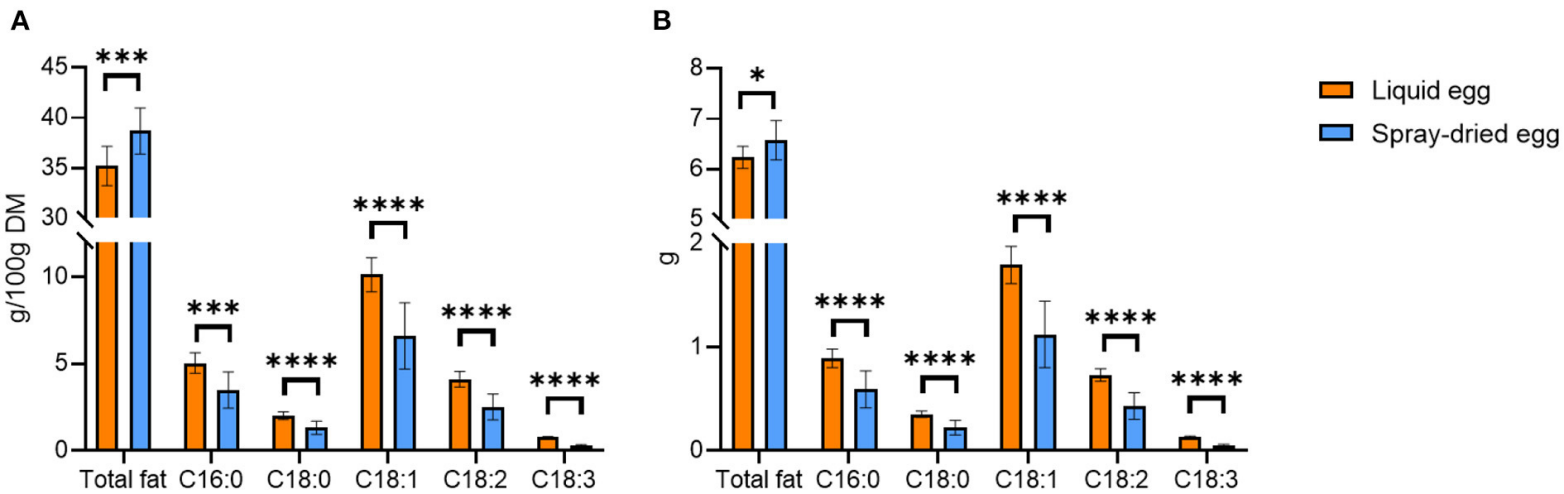
Average total fat content and fatty acid distribution of liquid eggs (orange bars) and egg powder (blue bars) in g/100 g DM **(A)** and g/egg **(B)**. Mean values ± SD (*n* = 12). Statistical significance between the egg powders and pasteurized whole egg displayed as: *p ≤ 0.05; ***p ≤ 0.001; ****p ≤ 0.0001.

The average total fat content of all pasteurized eggs analyzed was 11.3 ± 0.4 g/ 100 g whole egg (FW) or 35.2 ± 2.0 g/ 100 g DM, which amounts to 6.23 ± 0.22 g per egg. Spray-dried samples averaged at 38.7 ± 2.3 g/ 100 g DM or 6.57 ± 0.39 g per egg, showing a slight yet significant increase in the total fat content (Figure 1A).

Compared with the total fat content and fatty acid pattern from a previous analysis by Akpinar-Bayizit et al. (31), the concentrations obtained in the present study were in a similar range for both pasteurized and dried eggs. In detail, the average total fat content of liquid eggs determined by Akpinar-Bayizit et al. (31) was 10.04 g/ 100 g. This number corresponds well with that from the German Institute of Nutritional Information, reporting 9.3 g/ 100 g (32). After taking into account the drying factor of 3.84, a content of 38.6 ± 0.0 g/ 100 g can be calculated for Akpinar-Bayizit et al. (31), which would be slightly higher than the content determined in this study. The Souci-Fachmann-Kraut database states an average total fat content of 11.4 g/ 100 g FW (33), which is also in agreement with our values. For spray-dried eggs, Akpinar-Bayizit et al. (31) reported a mean total fat content of 40.4 ± 0.0 g/ 100 g, which was significantly higher than that of liquid whole eggs. It should be noted that the concentrations of the liquid egg products reported by Akpinar-Bayizit et al. (31) were compared with those of the spray-dried products without considering the drying factor, and no indication of the spray-drying temperature could be found for further clarification. That said, the higher fat content reported by these authors was probably largely due to a concentration during the drying process. Abreha et al. (18), who investigated the fat contents of spray-dried egg powder from eggs of two different chicken breeds, reported mean total fat contents of 36 g/ 100 g and 39 g/ 100 g. The recommendation of Lutter and Dewey (34) to meet a dietary intake of 6.3 g total fat by means of one daily portion of a fortified supplementary food (50 g) for infants aged 12–23 months, could be met by 71 and 77%, respectively, when 12.5 g of the whole egg powder is administered. With respect to the results presented in our study, consumption of 12.5 g whole egg powder would meet these recommendations by 71–80%.

Our analysis of fatty acids in pasteurized whole eggs yielded a homogenous distribution in all samples, with an average content of 10.1 ± 1.0 g/ 100 g and monounsaturated oleic acid accounting for 29% of total fatty acids. A higher proportion of saturated fatty acids (SFA, 19.4%) was analyzed compared to that of polyunsaturated fatty acids (PUFA, 13.9%). Palmitic acid was the most abundant saturated fatty acid, accounting approximately for 14% of the total fatty acids. Linoleic acid, an omega-6 fatty acid, was quantified with a mean content of 4.1 ± 0.5 g/ 100 g DM or 0.73 ± 0.06 g per egg, accounting for 11.6% of the total fatty acid content. The average concentration of the omega-3 fatty acid alpha-linolenic acid was 0.76 ± 0.05 g/ 100 g whole egg DM or 0.13 ± 0.01 g per egg, which corresponds to 2.5% of the total fatty acid content.

Oleic acid was also found to be the dominant fatty acid in spray-dried samples (Figure 1A). Nevertheless, in all egg powder samples, an average reduction of 34.8% compared to the liquid product was observed. The absolute contents shown in Figure 1B likewise highlighted greater losses for PUFAs than for SFAs. Linoleic acid decreased from 4.11 ± 0.45 to 2.52 ± 0.75 g/ 100 g DM and alpha-linolenic acid from 0.76 ± 0.05 to 0.30 ± 0.04 g/ 100 g DM. The discrepancy between similar total fat contents and losses of fatty acids after spray-drying can be explained by complete lipid extraction on one side and the selective quantification of specific compounds, deteriorated due to lipid oxidation, by GC-FID on the other. In the literature, reported by Stadelman and Cotterill (35), e.g., fat and fatty acid contents of the egg powder were often calculated only on the basis of the drying factor and not determined experimentally. Therefore, these values do not consider possible losses due to spray-drying. Javed et al. (36) examined fatty acid profiles using various spray-drying parameters on liquid and spray-dried eggs from hens fed with PUFA fortified designer feed. The authors reported slight DHA losses from 20.17 ± 0.67 mg/ 50 g egg to 17.65 ± 0.78 mg/ 50 g egg, showcasing that PUFA preservation can be achieved through optimization of the spray-drying process at least on a laboratory scale. In contrast, we could only record DHA in pasteurized liquid samples, with a mean DHA content of 120.5 ± 37.3 mg/ 100 g FW or 66.3 ± 6.3 mg per egg. All spray-dried egg samples were under the limit of quantification (<0.01 g/ 100 g), again confirming the high loss of PUFAs observed throughout this study.

Regarding the nutritional value, Figure 1B illustrates the absolute amounts of total fat content and fatty acids that can be delivered by pasteurized or spray-dried egg equaling one medium-sized egg. In detail, the intake of 55 g of fresh egg provides an average of 6.23 g of fat, of which 1.25 g are SFAs, 1.79 g monounsaturated (MUFAs) and 0.86 g PUFAs. The amount of 0.86 g PUFAs includes 0.73 g linoleic acid and 0.13 g alpha-linolenic acid. An adequate amount of dried egg (17 g) contains an average of 6.57 g of fat, of which 0.82 g accounts for SFAs, 1.12 g for MUFAs, and 0.48 g for PUFAs, the latter including 0.43 g of linoleic and 0.05 g of alpha-linolenic acid. Notably, as summarized in the conclusion of the Discussion section, for a selection of nutrients for infants aged 7–12 months and for children aged 1–8 years (**Figure 8**), these values meet the AI summarized by the National Academies Food and Nutrition Board (37) of linoleic acid by 9 and 5% and 10 and 6% for alpha linolenic acid, respectively.

### Peroxide value

Peroxide values of <10 meq O_2_/ kg fat for edible oils and fats, with the exception of up to 20 meq O_2_/ kg fat for virgin olive oil, are considered acceptable, while a higher value represents the progress of oxidation (38). Since eggs are also a source of dietary fat, this value was used as a reference point for lipid oxidation in the present study. For the pasteurized samples, an average peroxide content of 1.05 ± 0.17 meq O_2_/ kg fat (*n* = 19) could be determined. Spray-drying resulted in a significant increase to 1.68 ± 0.12 meq O_2_/ kg fat (*p* < 0.0001). Inflow temperature of 180°C led to a further increase, with 1.81 ± 0.06 meq O_2_/ kg fat.

In the study by Galobart et al. (39), the main cause of peroxide formation in dried eggs was attributed to the heat treatment. The larger surface area of the egg powder also provides a good surface for attack by oxygen and can thus start the radical chain reaction during storage or allow it to progress more rapidly. Therefore, special care was taken in our study during sample work-up, leaving as little head space as possible in the sample vessel. In order to minimize other influencing factors, such as storage time, peroxide analysis was performed immediately after sample collection. Lower values for egg powder were reported by Koç et al. (16), with 0.320–0.799 meq O_2_/ kg fat. Notably, Koç et al. (16) determined the peroxide value by a different analytical method, the official method according to Wheeler AOAC 2000 (28). Wheeler's method would require an amount of about 5 g of extracted fat. Since hen eggs have a fat content of approximately 10 g/ 100 g, a larger sample size would have been required to extract enough fat, which is why we chose the FOX assay for determination. Moreover, the study reported by Koç et al. (16) is lacking a comparison to pasteurized egg samples and is generally focused on optimizing the spray-drying process by preserving the functional properties while keeping the peroxide value low (16). The authors also showed that the outlet temperature had a significant effect on the peroxide value. The lowest peroxide value of 0.320 meq O_2_/ kg fat was obtained with 180°C inlet, 60°C outlet temperature and an atomization pressure of 392 kPa (16). Similarly, Javed et al. (36) examined the influence of various spray-drying and storage parameters on the peroxide value using a laboratory scale spray-dryer. The authors obtained peroxide values of 0.324 meq O_2_/ kg fat for normal eggs, 0.418 meq O_2_/ kg fat for PUFA fortified designer eggs and of 0.45–0.65 meq O_2_/ kg fat for spray-dried eggs, positively correlating with applied air temperatures. In addition, the authors reported significantly higher peroxide values when the samples were stored for up to 60 days at 25°C when compared to storage at 4°C. Nonetheless, in order to make a clear recommendation of the temperature conditions, it is also necessary to monitor other parameters, such as the changes in the concentrations of antioxidants in controlled studies.

### Amino acids

**Figure 2:**
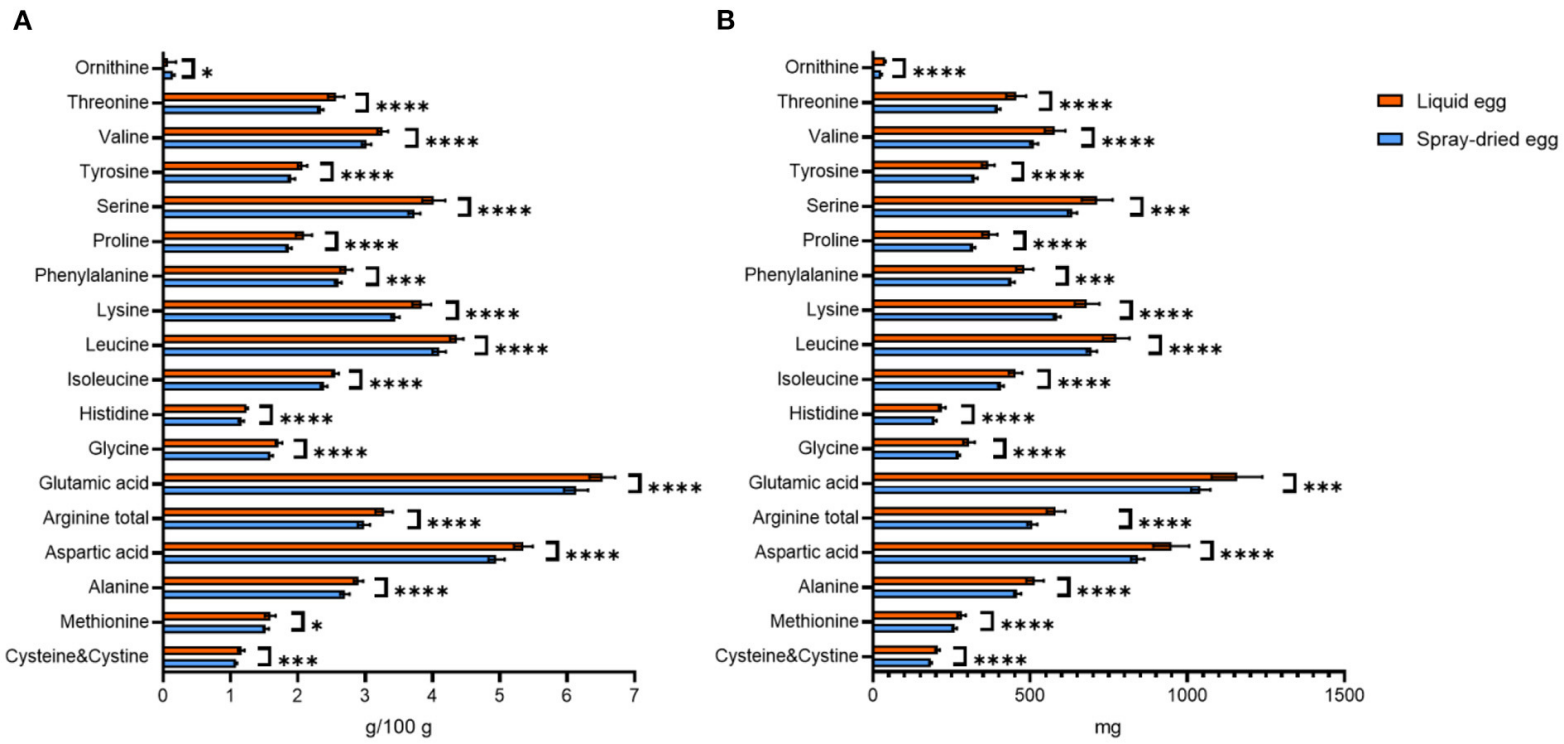
Contents of amino acids found in liquid eggs (orange bars) and egg powder (blue bars) in g/ 100 g **(A)** and mg per egg **(B)**. Results are presented as mean values ± standard deviation (*n* = 12). Statistical significance between the egg powders and pasteurized whole egg displayed as: *p ≤ 0.05; ***p ≤ 0.001; ****p ≤ 0.0001.

The fact that water-soluble nutrients generally were not influenced considerably by spray-drying also is reflected in our results of amino acids. Nonetheless, reductions due to spray-drying, especially in lysine content, have been reported for infant formula in the past (40), warranting in-depth analysis of other spray-dried foods. Even though reaching significance in most cases, the commercial egg processing investigated in our study did not result in great reductions (4–10%) in the content of lysine or other essential amino acids. In detail, the lysine content recorded for pasteurized eggs, 1.20 ± 0.09 g/ 100 g FW, is in agreement with USDA data on fresh whole egg (0.91 g/ 100 g) (30). Our result equals 3.86 ± 0.13 g/ 100 g DM, with 3.47 ± 0.13 g/ 100 g found for spray-dried eggs, thus only displaying a slight, however significant (*p* < 0.0001) reduction of 10%. In this regard, Vargas-del-Río et al. (41) recently demonstrated that the addition of sucrose prior to spray-drying could give further control over amino acid preservation. A complete comparison of amino acid contents before and after spray-drying is depicted in Figure 2.

Amino acid requirements and recommended dietary allowances (RDA) for selected amino acids are given in mg/ kg body weight per day in literature (42, 43). Hence, we used weight-for-age charts provided by the World Health Organization, choosing body weights of 7.6 kg for infants aged 6 months and 11.9 kg for 2 year old children (44). Converting the per egg values depicted in Figure 2B to the percent contribution to required intakes for both age groups, 17 g of spray-dried egg account for 100% each for isoleucine and valine, 100% and 80% for leucine or 100 and 77% for lysine, respectively. Thus, it can be concluded that eggs remain an excellent source of essential amino acids even after pasteurization and spray-drying.

In addition, we determined N(6)-Carboxymethyllysine (CML) contents to monitor slight reductions of essential amino acids caused by Maillard reactions during spray-drying. CML is used as marker for the increasingly studied group of advanced glycation end products (45, 46) and heat treatment has been reported to increase CML contents of egg white and yolk significantly (47). In accordance with the lysine measurement, however, we did not record a significant increase of CML contents due to spray-drying, with a value of 614.2 ± 142.2 mg/ 100 g DM for the spray-dried samples. Another marker for amino acid degradation, hydroxyproline, could not be detected in any of the samples (LOD = 0.05 g/ 100 g).

### Fat-soluble vitamins

#### Vitamin E

**Figure 3:**
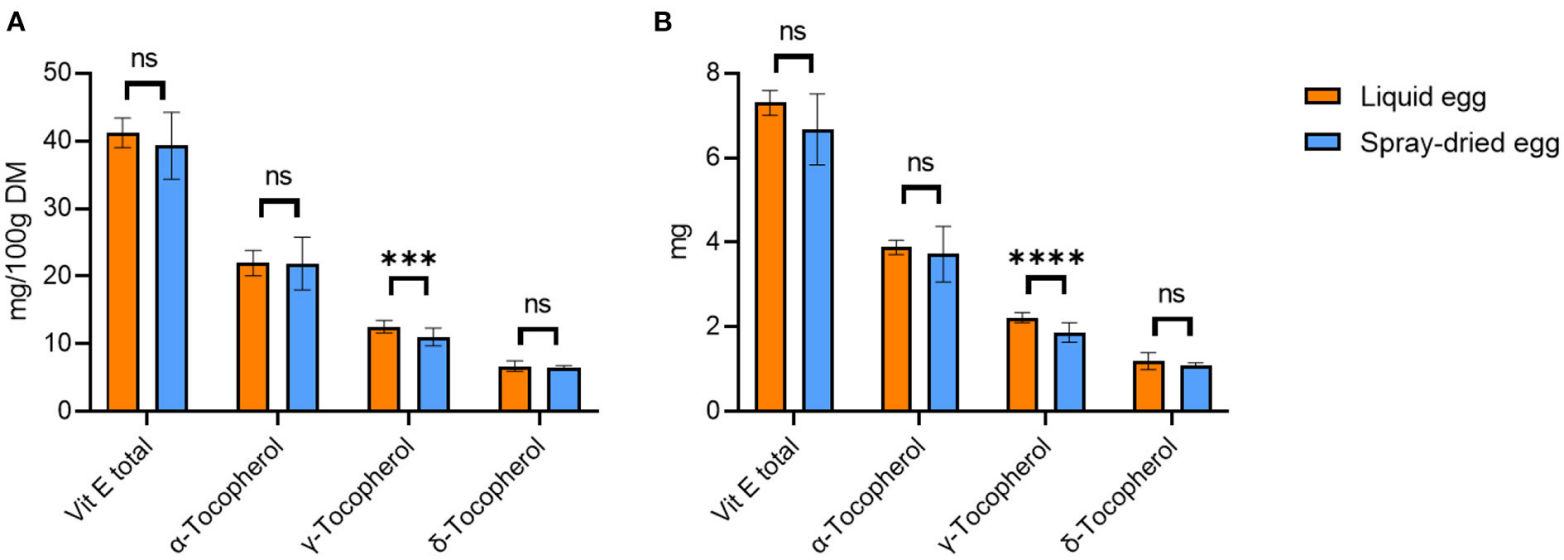
Tocopherol contents of liquid egg (orange bars) compared to spray-dried egg eggs (blue bars) in mg/ 100 g DM **(A)** and mg per egg **(B)**. Results are presented as mean values ± standard deviation (*n* = 15). Statistical significance between the egg powders and pasteurized whole egg displayed as: ns *p* > 0.05, *** *p* ≤ 0.001, **** *p* ≤ 0.0001.

Analysis of the pasteurized whole eggs revealed a mean total tocopherol content of 13.3 ± 0.6 mg/100 g FW, which corresponds to a mean content of 41.2 ± 2.2 mg/ 100 g DM. Quantitatively, α-tocopherol accounted for the largest proportion with 22.0 ± 1.9 mg/ 100 g DM. As shown in Figure 3A, spray-drying of eggs did not result in significant reductions of the overall tocopherol compounds, with mean total tocopherol contents of 39.3 ± 4.9 mg/ 100 g DM and 21.9 ± 3.9 mg/ 100 g DM for α-tocopherol. With regard to the discussed results of fatty acids, vitamin E plays an important role in preventing and delaying lipid oxidation, driven by the formation of oxygen radicals (48). This, however, could not be observed in our study. In detail, the oxygen radical scavenging ability is strongly dependent on the tocopherol homolog distribution (γ + δ)/α (48). Higher ratios such as a value of 4.77 reported for soybean oil lead to more pronounced delays in lipid oxidation and therefore higher storability when compared to canola oil (1.39) or sunflower oil (0.06) (48). The results obtained in our study yield ratios of 0.88 for liquid and 0.80 for spray-dried eggs, with the difference not reaching statistical significance. To counteract the peroxidation of PUFAs during production and storage, a higher vitamin E content in the feed should be considered for future optimizations (6).

Previously published data on the effect of the spray-drying process on the vitamin E content in eggs showed a wide range of results. In the analysis by Caboni et al. (20), no significant difference was found between pasteurized liquid eggs (20.4 mg/ 100 g DM) and spray-dried eggs (20.3 mg/ 100 g DM) with respect to α-tocopherol. Meynier et al. (19) reported slight losses of 10% during spray-drying at 160°C for α- and γ-tocopherol. In contrast, Galobart et al. (39) reported vitamin losses due to drying when applying similar spray-drying parameters as we did (160°C input and 90°C output temperature). The authors reported the highest vitamin E loss for eggs where hens had not received vitamin E fortified feed: compared to raw liquid eggs, the α-tocopherol content of the spray-dried egg powder was reduced by 48.2%. However, when the hens were supplemented with 50, 100, and 200 mg α-tocopherol acetate/kg feed, the α-tocopherol loss in spray-dried eggs was only 37.7, 33.5 and 31.1%, respectively. Worth mentioning is that Galobart et al. (39) reported storage in glass jars at room temperature until analysis: these conditions might have an impact on the results. How many days elapsed between spray-drying and analysis, or how much oxygenated headspace was present in the glass vessels used could not be determined from the publication. In contrast, we stored both pasteurized and spray-dried samples at −80°C until analysis, which might explain why we did not record a significant decrease in α-tocopherol. Apart from exposure to light, contact with oxygen and temperature also play a decisive role in vitamin E stability (49).

As depicted in Figure 3B, 55 g of pasteurized egg approximately provides 3.7 mg α-, 2.2 mg γ-, and 1.2 mg δ-tocopherol, whereas an equivalent amount of dried egg (17 g) provides 3.6 mg α-, 1.9 mg γ-, and 1.1 mg δ-tocopherol. Regarding the DRIs, 17 g of the spray-dried egg examined in this study would therefore completely cover the required daily intake for both infants and children, demonstrating an excellent nutritional source.

#### Vitamin A

**Figure 4:**
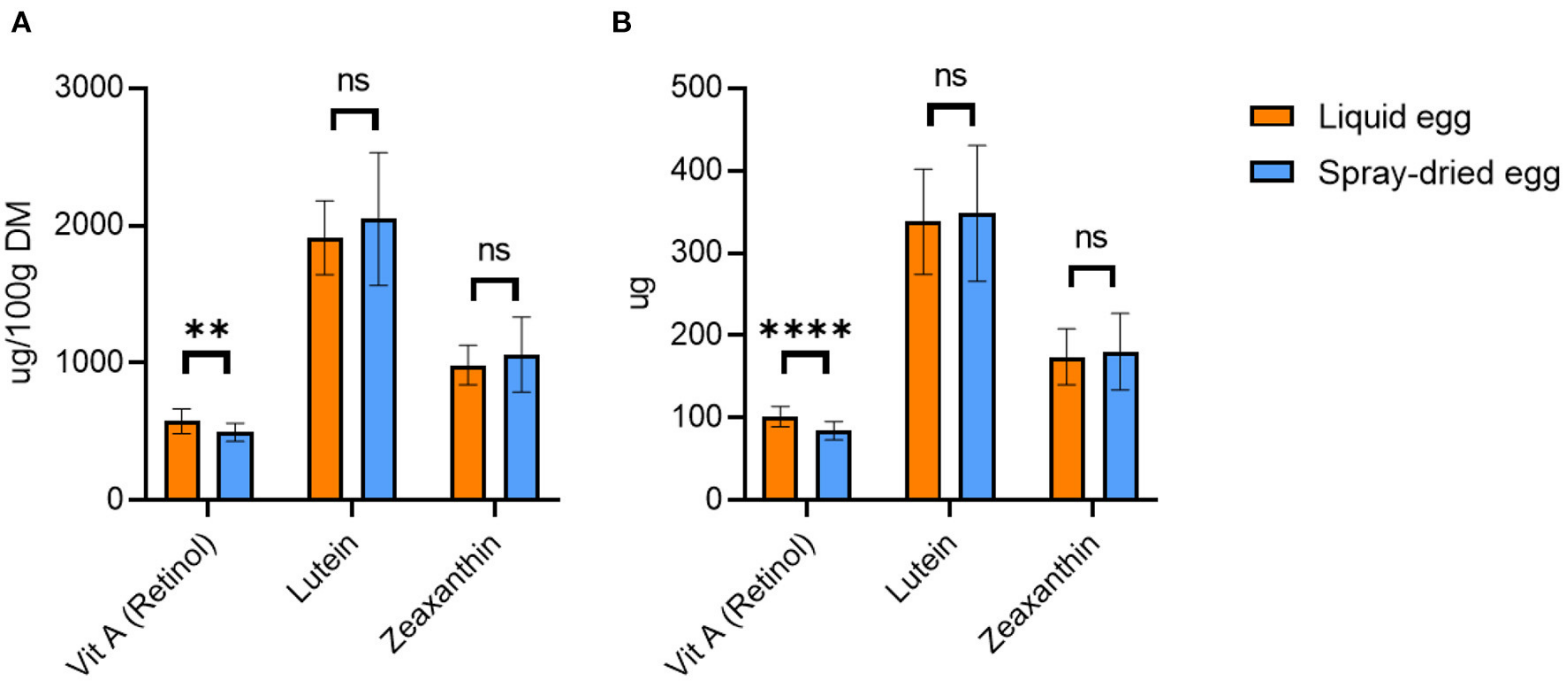
Retinol, lutein and zeaxanthin contents of liquid egg (orange bars) and egg powder (blue bars) in μg/ 100 g DM **(A)** and μg per egg **(B)**. Results are presented as mean values ± standard deviation (*n* = 18 for retinol, *n* = 6 for lutein and zeaxanthin). Statistical significance between the egg powders and pasteurized whole egg displayed as: ns *p* > 0.05, ** *p* ≤ 0.01, **** *p* ≤ 0.0001.

The retinol contents we determined, averaging at 184.9 ± 22.6 μg/ 100 g FW or 576.0 ± 89.7 μg/ 100 g DM (Figure 4A), are in agreement with those published by the DHSC (50), the USDA (51), and Miranda et al. (52) In detail, the DHSC reported an average vitamin A concentration of 120 μg/ 100 g in raw eggs, while Miranda et al. (52) noted a retinol content of 227 μg/ 100 g FW. The USDA, in turn, cited a mean retinol content in fresh eggs of 179 μg/ 100 g. These values are based on standard addition of vitamin A to the feed.

As shown in Figure 4A, spray-drying caused slight vitamin A losses to a mean value of 495.2 ± 66.2 μg/ 100 g DM, only reaching significance in two of the three batches. These variations between different batches suggest that spray-drying cannot be the sole cause of retinol loss. Generally, it is important to minimize oxidation-driving factors, such as oxygen or light, already at the feed level as well as during egg transport and storage. However, increasing the inflow temperature above 160°C proved unfavorable. We recorded a deterioration of 33.6% compared to the pasteurized whole egg sample for the 180°C batch.

To date, only a limited number of studies have investigated the effects spray-drying on the vitamin A content of eggs. Although the analysis by Caboni et al. (20) yielded an equally high total retinol content of 510 μg/ 100 g DM in pasteurized as well as spray-dried eggs, a more detailed observation showed a trans-cis shift of retinol. While pasteurized eggs had 440 μg/ 100 g of all-trans retinol content and 70 μg/ 100 g of cis retinol, this shifted to 420 μg/ 100 g of all-trans retinol and 90 μg/ 100 g of cis retinol in spray-dried eggs. This publication pointed out that a quantitatively detectable amount of cis-retinol isomers in the raw egg is rather uncommon and that this was probably due to differences in the metabolism of the chicken breed and/or occurrences in the feed. Notably, the lack of information regarding the spray-drying temperature limits a direct comparison with our results. Caboni et al. (20) also examined the influence of different storage temperatures. One-year storage of egg powder at 4°C did not result in a significant decrease in all-trans retinol, with a content of 380 μg/ 100 g DM. In contrast, storage at 20°C during the same period caused a decrease to 170 μg/ 100 g DM (20). These results highlighted that the storage temperature of the egg powder is quite relevant for the preservation of vitamin A. Although the shelf life of dried egg is definitely longer compared to fresh egg, a timely consumption should still be aimed at in order to avoid losses of vitamin A.

Figure 4B illustrates the amount of retinol ingested with 55 g pasteurized egg and 17 g spray-dried egg, respectively. While pasteurized egg contains an average of 101.7 μg retinol, a similarly high value of 84.2 μg can be achieved by the intake of 17 g powder. Thus, the recommended DRI for infants and children can be met by 17 and 24% respectively, again showcasing the high potential of spray-dried egg as a viable source of nutrients.

#### Carotenoids

Figure 4A also contains the mean content of important carotenoids lutein and zeaxanthin, with 1.9 ± 0.3 and 1.0 ± 0.1 mg/ 100 g DM for pasteurized and 2.0 ± 0.5 and 1.1 ± 0.3 mg/ 100 g DM for spray-dried eggs, respectively. This amounts to 348 μg lutein and 180 μg zeaxanthin after consumption of 17g of spray-dried egg (Figure 4B). Hence, no significant change in their concentrations after spray-drying was observed in any of the three batches investigated. Previous studies came to different conclusions in this regard. A temperature of 61.5°C and a pasteurization time of 3.5 mins did not cause significant changes in lutein and zeaxanthin concentrations in the study by Wenzel et al. (53) However, significantly higher xanthophyll contents were determined after spray-drying at 72°C. Lutein and zeaxanthin increased respectively from 164.9 ± 3.2 μg/ 100 g (FW) whole egg to 210.4 ± 8.2 μg/ 100 g (FW) dry egg and from 102.4 ± 1.9 μg/ 100 g whole egg (FW) to 128.9 ± 6.7 μg/ 100 g (FW) dry egg. The authors attributed this result to the structural unfolding of lipoproteins and destabilization of the cell matrix caused by heat exposure. The lipids of the lipoproteins, which are associated with lutein and zeaxanthin, could thus be cleaved off, increasing the extractability of xanthophylls. However, compared to other studies, such as that of Caboni et al. (20), the authors chose a temperature of 72°C, which is uncharacteristically low for spray-drying and could be one reason why the carotenoids were largely retained. Moreover, there was no information on the duration of spray-drying or other conditions during processing. Another study by Meynier et al. (19) found no effect of pasteurization or of spray-drying at 160 and 180°C on lutein and zeaxanthin content in eggs from a control group of hens without feed fortification.

#### Vitamin D_3_ and K_1_

**Figure 5:**
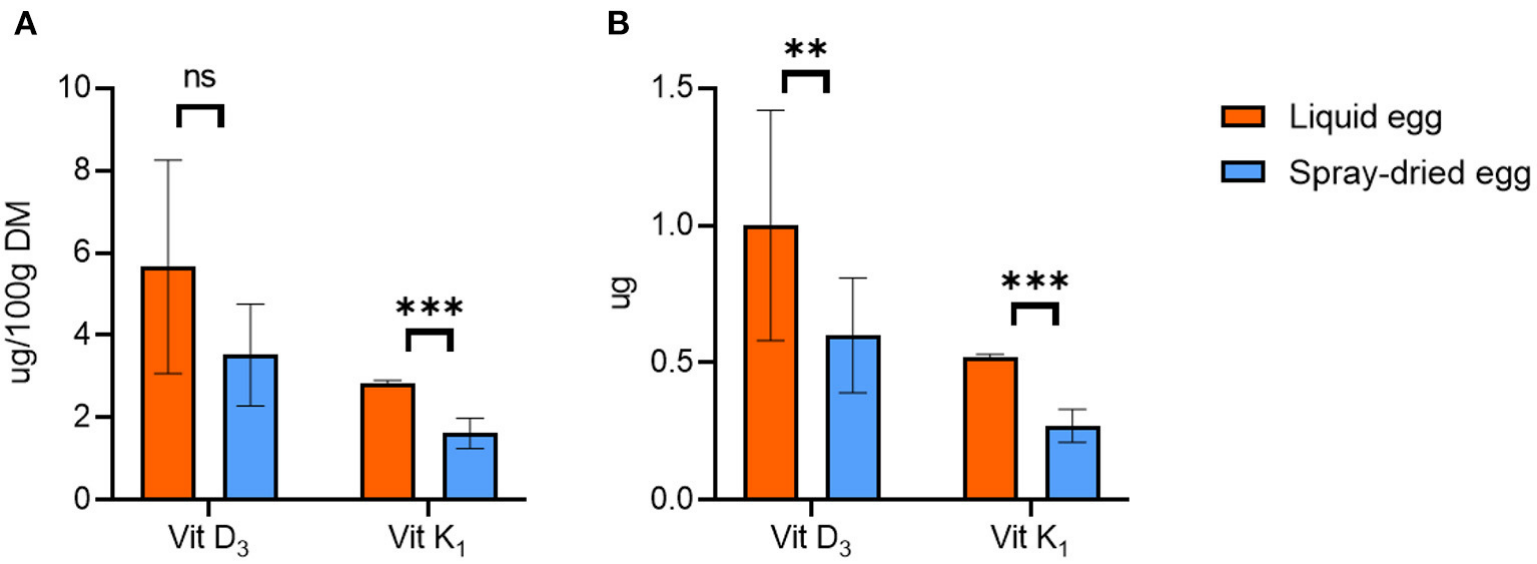
Contents of cholecalciferol (vitamin D_3_) and phytomenadione (vitamin _K1_) in pasteurized egg (orange bars) and egg powder (blue bars) in **(A)** μg/ 100 g dry matter and **(B)** μg per egg. Results are presented as mean values ± standard deviation (*n* = 12 for vit D_3_ and *n* = 6 for vit K_1_). Statistical significance between the egg powders and pasteurized whole egg displayed as: ns *p* > 0.05, **p ≤ 0.01, ***p ≤ 0.001. To display the effect of spray-drying on vitamin K_1_ content, only samples with contents in pasteurized eggs above the LOQ of 0.8 μg/100 g were considered for illustration.

The mean values of cholecalciferol (vitamin D_3_) in DM were 5.7 ± 2.7 μg/ 100 g for pasteurized and 3.5 ± 1.2 μg/ 100 g for spray-dried samples (Figure 5), which corresponds to a reduction of 33.8% due to further processing. However, homogenous results were only obtained for one batch, which is reflected in the otherwise high standard deviations and the difference not reaching significance based on g/ 100 g egg calculations. This observation can also be seen in databases, e.g., of the USDA, where values of whole eggs range from 0 to 8.19 μg/ 100 g, averaging at 2.46 μg/ 100 g (51). Only comparing the batch with more homogenous results within the subsamples yielded a reduction of 15.3%. Slight losses of 7% vitamin D during spray-drying of eggs have been reported as early as 1944 (54), and in general, vitamin D loss during food processing is a commonly known problem (55).

Concerning phytomenadione (vitamin K_1_), overall comparisons are impeded as only three subsamples from two batches (*n* = 6) of the pasteurized eggs produced values above the LOD of 0.8 μg/ 100 g, resulting in 0.94 ± 0.01 μg/ 100 g FW or 2.84 ± 0.06 μg/ 100 g DM. The spray-dried samples all exceeded the LOD and averaged at 1.62 ± 0.37 μg/ 100 g DM (Figure 5A). This is in accordance with a content of 1.2 μg/ 100 g DM for dried eggs given by the USDA (56). Nonetheless, in the few cases where comparisons are possible, the reduction due to spray-drying ranged from 28 to 54%. Phytomenadione is repeatedly reported as being heat-stable and retained after most cooking processes (57), and at least for cow's milk, this could be shown for spray-drying as well (58). Hence, the overall low values we recorded cannot be explained entirely by processing of the eggs and may be accounted to the high photosensitivity of the compound. However, even contents for whole fresh eggs vary greatly in databases, ranging, for example, from 0.3 μg/100 g (30) to 8.9 μg/ 100 g (33).

#### Vitamin B group

**Figure 6:**
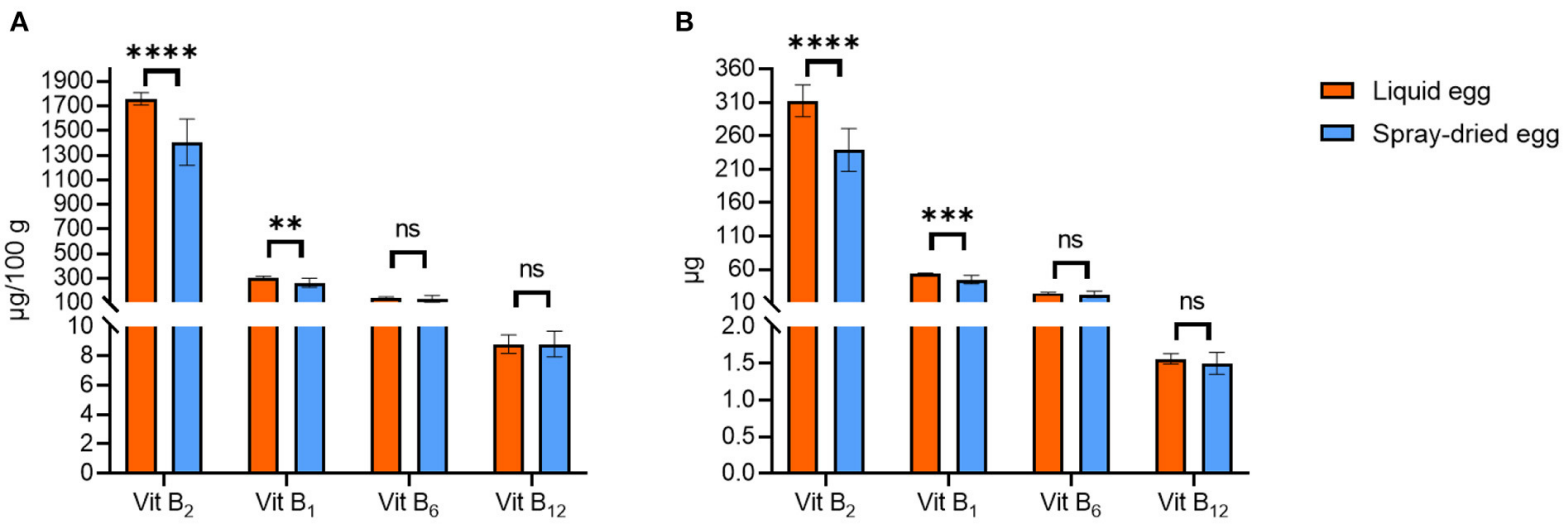
Contents of vitamin B compounds in pasteurized egg (orange bars) and egg powder (blue bars) in μg/ 100 g DM **(A)** and μg per egg **(B)**. Results are presented as mean values ± standard deviation (*n* = 12). Statistical significance between the egg powders and pasteurized whole egg displayed as: ns *p* > 0.05, ** *p* ≤ 0.01, *** *p* ≤ 0.001, ****p ≤ 0.0001.

Figure 6A illustrates the contents of several vitamin B compounds examined in this study. Generally, as we could demonstrate for other analytes, values for pasteurized eggs are well within reported ranges for fresh whole eggs. For instance, the fresh weight mean value for riboflavin (vitamin B_2_) was 567.7 ± 43.1 μg/ 100 g, with 408 μg/ 100 g being reported in SFK (33) or 410–520 μg/ 100 g by the USDA for raw whole eggs (30).

Riboflavin was the only B vitamin for which a noteworthy reduction during spray-drying could be observed, from 1.76 ± 0.05 to 1.41 ± 0.19 mg/ 100 g DM. Similar results have been reported before, e.g., 1.36 mg/ 100 g DM by Cotterill et al. (59) A direct comparison between pasteurized and dried eggs, where no effect of processing was reported, dates back to 1944 in a study by Denton et al. (54), in which values before and after drying were identical to the contents we obtained, with 1.3–1.4 mg/ 100 g DM. Similar to phytomenadione, riboflavin is considered to be heat stable but quite photosensitive, with average losses in milk as high as 80% after 2 h in light, which may be an explanation for study results where contents before and after treatment differ significantly or contents are low throughout (57). Regarding DRI, spray-dried eggs meet the recommended contents exceptionally well—in the case of infants aged 7–12 months by 100% for B_12_, by 60% for B_2_, by 15% for B_1_ and by 8% for B_6._ In sum, spray-drying does not change the status of eggs as important vitamin B sources, and as drying has been deemed crucial to retain this high nutritional value in storage examinations, e.g., for thiamin, (60) spray-drying represents an adequate choice.

### Mineral and trace element composition

**Figure 7:**
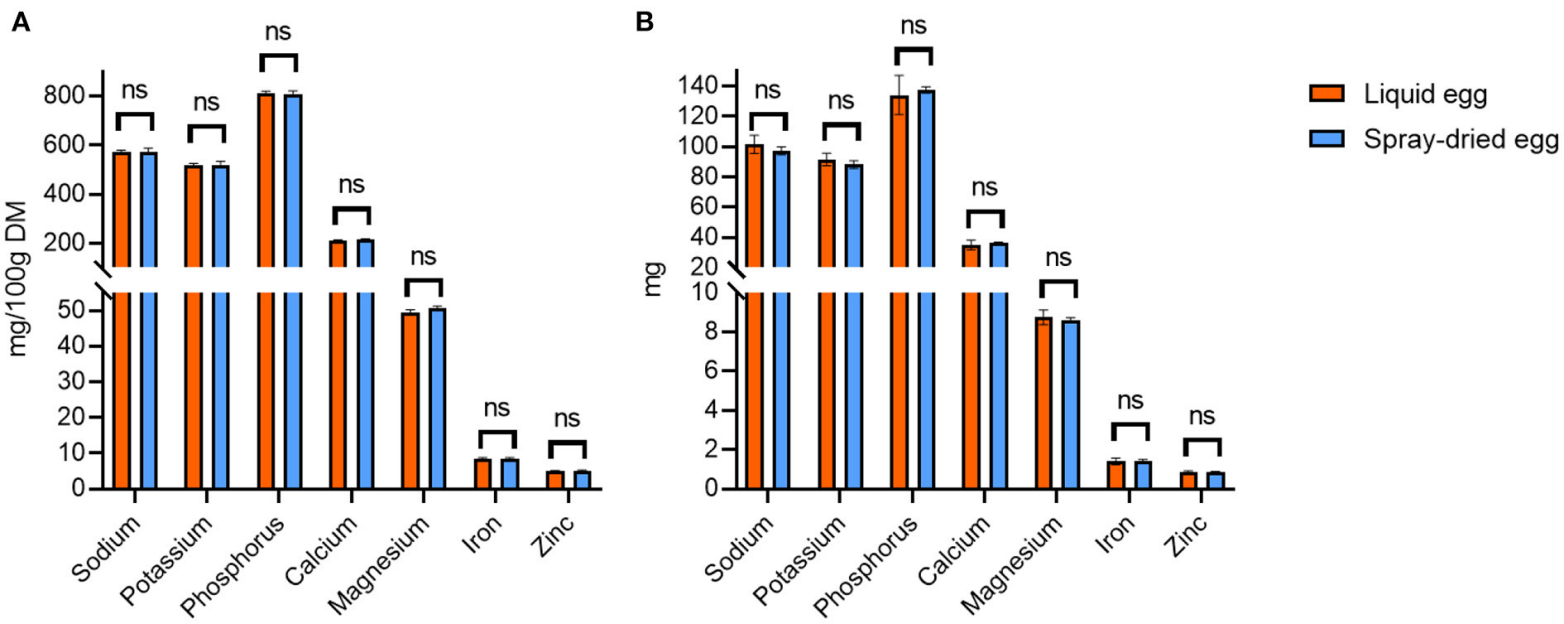
Selection of nutritionally relevant elements contained in liquid eggs (orange bars) and egg powder (blue bars) in mg/ 100 g **(A)** and mg per egg **(B)**. Results are presented as mean values ± standard deviation (*n* = 12). No statistical significant differences were found.

ICP-MS measurements were carried out to examine the overall mineral composition of both products concerning nutritional quality and safety, as well as to compare possible losses of nutritive elements or concentration of potentially toxic non-essential trace elements during spray-drying. Regarding the former, data of the mineral and trace element composition of both products are in accordance with the respective data given by the USDA for whole, pasteurized (61) and dried egg (56). Contents for a selection of nutritionally relevant elements are illustrated in Figure 7A. The analysis generally yielded homogenous results between all batches with no significant differences between the respective samples before and after spray-drying. Regarding the per egg intake depicted in Figure 7B, 17 g of spray-dried egg contained, e.g., 1.43 ± 0.07 mg iron and 0.88 ± 0.02 mg zinc. These values meet DRI for infants and children by 13 and 17% for iron and 29 and 22% for zinc, respectively, thus showing a high potential to aid in achieving the required total amounts. Two other noteworthy results not included in Figure 7 regard selenium and copper, of which 17 g spray-dried egg powder contain 15.1 ± 0.2 and 46.7 ± 6.5 μg, respectively, which amounts to 77 and 21% of the adequate intake for infants aged 7–12 months (37, 62).

In addition, a contamination with high levels of non-essential, potentially harmful trace elements mercury, lead, cadmium and arsenic can be excluded. Levels were well below the respective threshold values defined by the European Commission (63) and, except for Pb, under the level of quantification enabled by the highly sensitive technique in almost all cases. In detail, Pb levels in the egg powder samples were 7.74 ± 4.77 μg/ kg DM, whereas the threshold value of the European Commission for infant food marketed as powder is 20 μg/ kg FW (64), therefore approximately averaging to a tenfold ratio between our samples and the cutoff.

Hence, it can be summarized that both product groups, pasteurized whole eggs as well as spray-dried eggs, can be considered safe concerning non-essential, potentially harmful trace elements and that their consumption, even when applied as complementary food for infants, would not significantly contribute to overall heavy metal exposure. In addition, the high nutritional quality of eggs regarding their mineral and trace element content could be preserved in spray-dried eggs. These results further recommend a larger scale promotion of egg powder to prevent infant malnutrition in developing countries, as complementary food preparations in this sector have been reported to often lack essential elements such as zinc or iron (65).

### Nutritional quality

**Figure 8:**
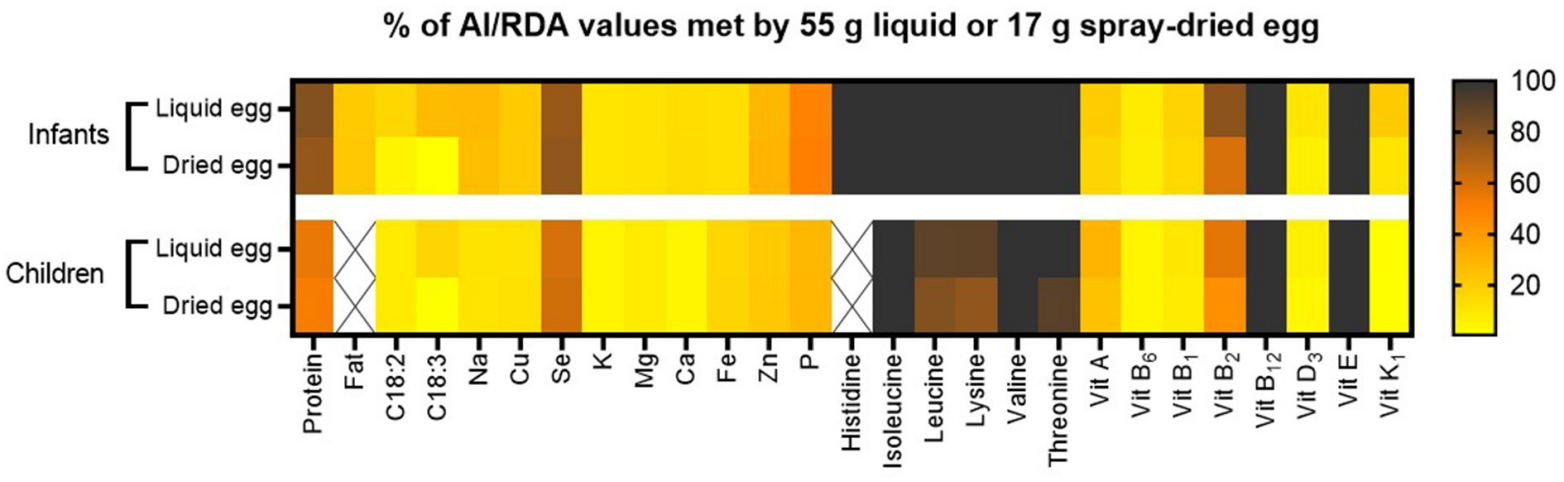
Percent of respective AI and RDA values met by consumption of 55 g pasteurized whole egg or 17 g spray-dried egg powder. RDA values for amino acids were calculated for infants aged 6 months with an average weight of 6.4 kg and children aged 2 years with an average weight of 11.9 kg. Other RDA and AI values used are estimations for infants aged between 7 and 12 months and children between 1 and 8 years. Nutrients without respective reference value for this population are crossed out.

Figure 8 summarizes the nutritional quality of both pasteurized whole egg and spray-dried egg, illustrating by which percentage 55 g of pasteurized whole egg or 17 g of egg powder meet AI (acceptable intakes) or RDA values of selected nutrients.

As discussed in the respective subsections, spray-drying could mostly retain the high nutritional quality of pasteurized whole eggs without accumulating potentially harmful compounds. Moreover, the potential of eggs in helping to prevent malnutrition in vulnerable population groups is further enhanced by their possible use in spray-dried, concentrated form, with easier storability, which both represent crucial factors for the application in low- and middle-income countries. Whereas the commercial spray-drying process with an inflow temperature of 160°C and outflow between 80 and 90°C proved to be an apt choice for the majority of nutrients, such as vitamins E, B or essential amino acids, the significant deterioration of unsaturated fatty acids demonstrated that the process still requires optimization. This is evident in content losses of 38.7% for linoleic and 60.8% for linolenic acid. Increasing antioxidant vitamin E levels in the feed could potentially prevent high PUFA losses during drying. For infants, the consumption of 55 g pasteurized whole egg meets the AI of α-linolenic acid by 27%, which represents a desirable amount and benchmark for future adaptations to spray-drying. In this regard, published data on egg powder produced from designer eggs in a laboratory scale spray-dryer (36) could already reveal that the combination of PUFA supplemented chicken feed and optimized spray-drying conditions can yield egg powder significantly richer in PUFAs than normal eggs. These results now need to be translated to industrial scale, combining effective feed supplementation with LCPUFAs (EPA and DHA) as well as vitamins E, A and D, with the preservation of all nutrients highlighted in our study, thus exploiting the full potential of spray-dried eggs as nutritional supplement.

## Data availability statement

The original contributions presented in the study are included in the article/Supplementary material, further inquiries can be directed to the corresponding author.

## Author contributions

BS, KK, and VS designed research. PP, SG, AD, and MP conducted research and analyzed data. PP, SG, and VS wrote the paper. VS had primary responsibility for final content. All authors read and approved the final manuscript.

## Conflict of interest

Author BS was employed by OVOBEST Eiprodukte GmbH & Co. KG. The remaining authors declare that the research was conducted in the absence of any commercial or financial relationships that could be construed as a potential conflict of interest.

## Publisher's note

All claims expressed in this article are solely those of the authors and do not necessarily represent those of their affiliated organizations, or those of the publisher, the editors and the reviewers. Any product that may be evaluated in this article, or claim that may be made by its manufacturer, is not guaranteed or endorsed by the publisher.
