# Supplementary material for: Evaluation of spray-dried eggs as a micronutrient-rich nutritional supplement
Source: Front Nutr. 2022 Sep 2;9:984715. doi: 10.3389/fnut.2022.984715 (PMC9479153; doi:10.3389/fnut.2022.984715)
Supplement: Supplementary file 1 [file Data_Sheet_1.docx]

Supplementary Material

Evaluation of spray-dried eggs as a micronutrient-rich nutritional supplement

Philip Pirkwieser ^1^, Silke Grosshagauer ^2^, Andreas Dunkel ^1^, Marc Pignitter ^2^, Bernard Schneppe^3^, Klaus Kraemer ^4,5^ and Veronika Somoza ^1,2,6*^

^1^ Leibniz Institute for Food Systems Biology at the Technical University of Munich, Freising, Germany

^2^ Department of Physiological Chemistry, Faculty of Chemistry, University of Vienna, Vienna, Austria,

^3^ OVOBEST Eiprodukte GmbH & Co. KG, Neuenkirchen-Vörden, Germany

^4^ Sight and Life Foundation, Basel,Switzerland

^5^ Department of International Health, Johns Hopkins Bloomberg School of Public Health, Baltimore, MD, United States

^6^ Chair of Nutritional Systems Biology, School of Life Sciences Weihenstephan, Technical University of Munich, Freising, Germany*****

**Correspondence:**Veronika Somoza
[v.somoza.leibniz-lsb@tum.de](mailto:v.somoza.leibniz-lsb@tum.de)

Table S1: Summary of determined analytes calculated per 100 g sample for pasteurized whole egg fresh weight (FW) as well as the calculated dry matter (DM) contents and measured DM contents for spray-dried egg powder.

Table S2: Summary of determined analytes calculated per egg, using 55g pasteurized whole egg and 17 g spray-dried egg powder.

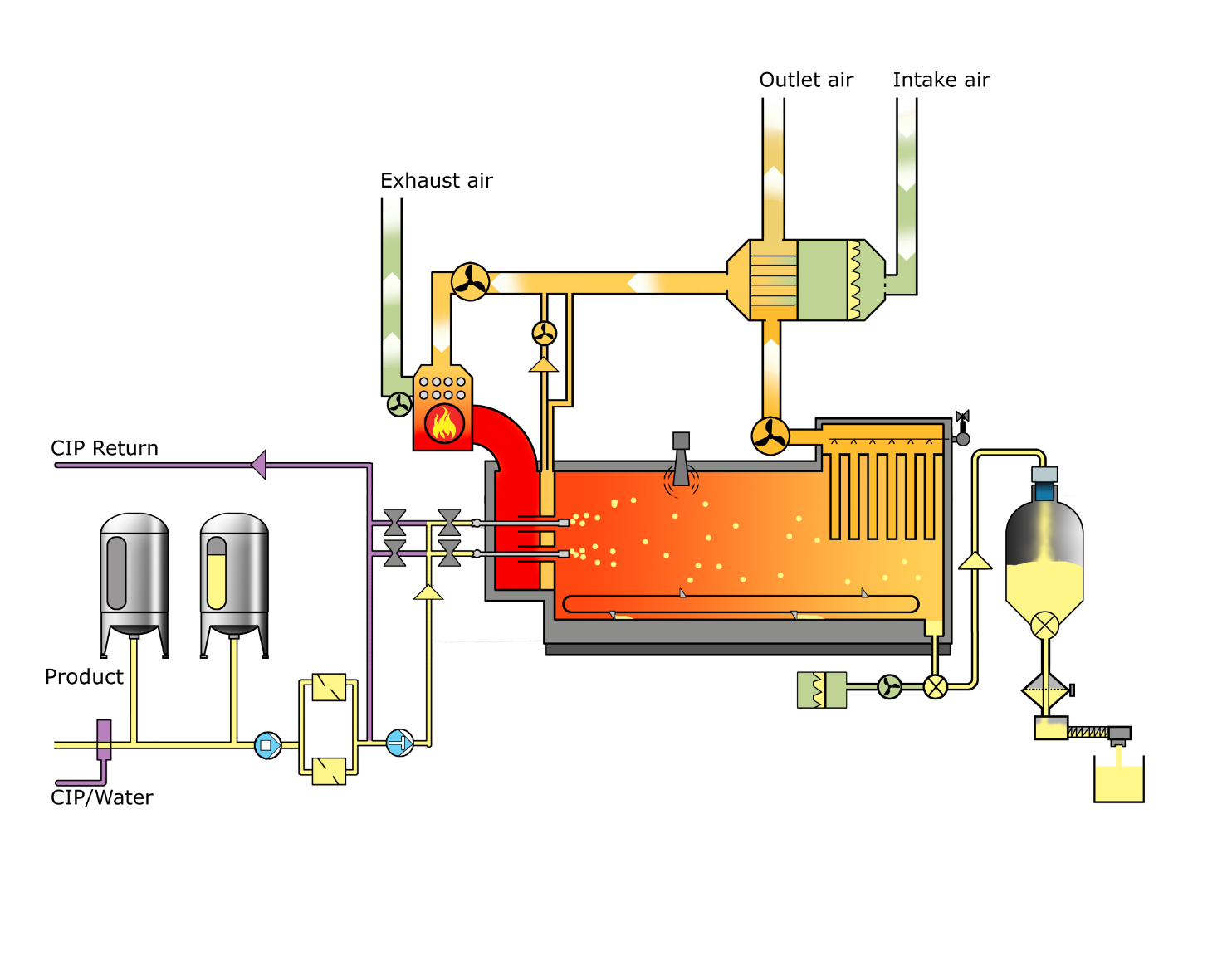


Figure S1: Simplified flow sketch of a box spray dryer, kindly provided by Sanovo Technology Group.
